# Investigation of the link between fluid shift and airway collapsibility as a mechanism for obstructive sleep apnea in congestive heart failure

**DOI:** 10.14814/phy2.12956

**Published:** 2017-01-05

**Authors:** Tom Carlisle, Neil R. Ward, Angela Atalla, Martin R. Cowie, Anita K. Simonds, Mary J. Morrell

**Affiliations:** ^1^ Academic Unit of Sleep and BreathingNational Heart and Lung InstituteImperial College LondonLondonUK; ^2^ NIHR Respiratory Biomedical Research Unit at the Royal Brompton and Harefield NHS Foundation TrustImperial College LondonLondonUK; ^3^ NIHR Cardiovascular Biomedical Research Unit at the Royal Brompton and Harefield NHS Foundation TrustImperial College LondonLondonUK

**Keywords:** Heart failure, obstructive sleep apnea, pcrit, pharyngeal collapsibility, rostral fluid shift

## Abstract

The increased prevalence of obstructive sleep apnea (OSA) in congestive heart failure (CHF) may be associated with rostral fluid shift. We investigated the effect of overnight rostral fluid shift on pharyngeal collapsibility (Pcrit), pharyngeal caliber (APmean), and apnea‐hypopnea index (AHI) in CHF patients. Twenty‐three optimally treated systolic CHF patients were studied. Neck circumference was measured immediately prior to sleep in the evening and immediately after waking in the morning as a marker of rostral fluid shift. Pcrit was measured during sleep, early and late in the night. APmean was measured using acoustic reflection at the same times as neck circumference measurements. 15/23 CHF patients experienced an overnight increase in neck circumference; overall neck circumference significantly increased overnight (mean±SD, evening: 41.7 ± 3.2 cm; morning: 42.3 ± 3.1 cm; *P* = 0.03). Pcrit increased significantly overnight (early‐night: −3.8 ± 3.3 cmH_2_O; late‐night: −2.6 ± 3.0 cmH_2_O; *P* = 0.03) and APmean decreased (evening: 4.2 ± 1.3 cm^2^; morning: 3.7 ± 1.3 cm^2^; *P* = 0.006). The total AHI correlated with neck circumference (*r* = 0.4; *P* = 0.04) and Pcrit (*r* = 0.5; *P* = 0.01). APmean correlated with neck circumference (*r* = −0.47; *P* = 0.02). There was no significant change in AHI between the first and second half of the night (first‐half: 12.9 ± 12.4/h; second‐half: 13.7 ± 13.3/h; *P* = 0.6). Overnight rostral fluid shift was associated with increased pharyngeal collapsibility and decreased pharyngeal caliber during sleep in CHF patients. Rostral fluid shift may be an important mechanism of OSA in this patient group.

## Introduction

The prevalence of obstructive sleep apnea (OSA) is increased in patients with congestive heart failure (CHF) to approximately 25% (Javaheri et al. 1998; Lanfranchi et al. 2003; Ferrier et al. 2005; Javaheri 2006; Oldenburg et al. 2007; Schulz et al. 2007; Vazir et al. 2007; MacDonald et al. 2008; Bitter et al. 2009), compared to approximately 14% in the general population aged 50–60 years (Young et al. 1993; Bixler et al. 1998, 2001; Durán et al. 2001; Peppard et al. 2013). A feature of CHF that may contribute to the increased prevalence of OSA is peripheral and pulmonary edema. It is postulated that the edema, which accumulates during the day, moves rostrally overnight; resulting in extraluminal pressure being exerted on the pharyngeal airway (White and Bradley 2013). The increased transmural pressure and attenuation of the upper airway muscle reflexes (Horner et al. 1994) could result in pharyngeal collapse and OSA.

Rostral fluid shift as a mechanism for pharyngeal collapse was first proposed by Shepard et al. (1996). They speculated that an increase in fluid surrounding the pharynx could result in increased extraluminal pressure. Both phenomena have been demonstrated experimentally by actively inducing fluid shift using lower body positive pressure in awake healthy volunteers (Chiu et al. 2006; Shiota et al. 2007; Su et al. 2008). Pharyngeal narrowing has also been demonstrated in awake OSA patients (White et al. 2014). However, results have been more equivocal when examining spontaneous overnight fluid shift. In OSA patients without CHF, an overnight increase in neck circumference was not observed in all patients (Jafari and Mohsenin 2011), and in those that did experience an overnight increase in neck circumference, this was not associated with the severity of OSA, nor with an overnight increase in the apnea‐hypopnea index (AHI) (Jafari and Mohsenin 2011; Fischer et al. 2012). In CHF patients with OSA, and CHF patients with central sleep apnea (CSA), an overnight decrease in leg fluid volume has been found to correlate with the AHI (Yumino et al. 2010). However, to the best of our knowledge, no measurements of pharyngeal collapsibility or pharyngeal caliber have been reported in CHF patients, nor have these measurement been made across the night.

The aim of this study was to investigate the effects of overnight rostral fluid shift on pharyngeal collapsibility in CHF patients. Neck circumference was taken as a marker for rostral fluid shift, as in previous studies (Chiu et al. 2006; Shiota et al. 2007; Su et al. 2008, 2009; Redolfi et al. 2009, 2011a,b; Yumino et al. 2010; Jafari and Mohsenin 2011; Elias et al. 2012; Fischer et al. 2012; White et al. 2014) and was measured before and after sleep in the supine posture. The primary outcome measure was pharyngeal collapsibility, measured using pharyngeal critical closing pressure early and late in the night during non‐REM sleep. Secondary outcome measures included pharyngeal caliber, measured using acoustic reflection (Hilberg and Pedersen 2000) before and after sleep, and the AHI in the first and second half of the night. We tested the primary hypothesis that the pharynx would be more collapsible in the late part of the night compared to the early part of the night in CHF patients with rostral fluid shift. We also hypothesized that the pharyngeal caliber would decrease overnight and the AHI would increase overnight.

## Methods

### Patients

Patients with systolic CHF (New York Heart Association II–III) were recruited from heart failure clinics at the Royal Brompton Hospital. Eligible patients were clinically stable, with no CHF‐related hospital admissions 3 months prior to recruitment. Exclusion criteria were any sleep disorder other than OSA including >50% central sleep apnea, previous upper airway surgery, or current treatment for sleep apnea. Females were not excluded from this study, however, all volunteers were male. The Brompton Harefield and NHLI Research Ethics Committee approved the study and all participants gave written informed consent.

Patients attended the sleep laboratory on two occasions, no more than 1 month apart. On the first visit, a diagnostic nocturnal polysomnography (nPSG) screened for sleep apnea, and measurements of neck circumference, pharyngeal caliber, and weight were made before and after sleep. Neck circumference and pharyngeal caliber were measured in the supine posture. Weight was measured in the evening, immediately before patients went to sleep, and in the morning after they had woken up, before passing urine or consuming any food or drink. Demographic and clinical information were recorded. On the second visit, measurements of pharyngeal collapsibility were made during sleep using the passive pharyngeal critical closing pressure (Pcrit) technique (Schwartz et al. 1998).

### Neck circumference

Neck circumference was measured in the supine posture before sleep between 2000 and 2200 h, and after waking from sleep between 0600 and 0800 h. Before each set of measurements, participants rested quietly for 5 min. Neck circumference was measured below the cricothyroid cartilage (to the nearest mm). Marks were made on the skin after the first measurement to ensure that subsequent measurements in the morning were made at the same level.

### Pharyngeal collapsibility

Pharyngeal collapsibility (Pcrit) can be measured under conditions of active neuromuscular tone (active Pcrit) (Schwartz et al. 1988) or passive neuromuscular tone (passive Pcrit) (Schwartz et al. 1998). Active Pcrit is thought to reflect the effect of the dynamic neuromuscular response to upper airway resistance on collapsibility, whereas passive Pcrit is thought to reflect the effect of mechanical loads on collapsibility (Patil et al. 2007). In this study, we measured pharyngeal collapsibility using the passive Pcrit technique as rostral fluid shift would be expected to exert a mechanical challenge to pharyngeal patency.

During Pcrit measurements, patients slept connected to a breathing circuit capable of delivering nasal pressures between +15 cmH_2_O (Companion 318, Puritan Bennett, Colorado) and −15 cmH_2_O (Pegaso Cough, Dima Italia, Italy). The pressure was rapidly switched using a manual three‐way tap. Airflow was monitored using a pneumotachometer (Model 4700A, Hans Rudolph Inc., Kansas City, MO). Respiratory effort was monitored using uncalibrated respiratory inductance plethysmography, and in those who were able, an esophageal pressure cannula (CTO‐1, plus S7b/2 pressure transducer, Gaeltec Devices Ltd, Dunvegan, UK) was positioned using standard procedures (Morrell et al. 1995).

Patients were permitted to sleep in a supine or lateral posture. Pcrit measurements were performed during stage N2 or N3 sleep, early (within the first sleep cycle) and late (approximately 4 h after the early measurement) in the night. Outside of Pcrit measurements, patients were left connected to the breathing circuit with minimal CPAP, to prevent CO_2_ accumulation in the breathing circuit (~3 cmH_2_O). Once patients had entered stage N2 or N3 sleep, CPAP was titrated to eliminate flow limitation (holding pressure). The mean holding pressure was 4.8 ± 1.4 cmH_2_O (range 3–9 cmH_2_O). After at least 3 min of stable sleep, mask pressure was rapidly reduced from holding pressure for 3–5 breaths, and then returned to holding pressure. After at least 1 min, the pressure was reduced further for 3–5 breaths. This was repeated, reducing the mask pressure in 1 or 2 cmH_2_O increments until airflow ceased accompanied by ongoing esophageal pressure swings, or ongoing thoracic and abdominal movements (in those patients unable to tolerate the esophageal pressure cannula). In some patients, it was not possible to reach a pressure that caused airflow to cease without producing an arousal from sleep. In these patients, linear regression was performed to predict the pressure at which airflow would have ceased (Patil 2004).

### Pharyngeal caliber

Pharyngeal caliber was measured during wakefulness in the supine posture, in the evening and morning immediately after neck circumference measurements. Pharyngeal caliber was measured noninvasively with acoustic reflection (A1 Executive Acoustic Rhinometer; GM Instruments Ltd, Kilwinning, UK) using standard techniques (Hilberg and Pedersen 2000) and protocols described by our group previously (Carlisle et al. 2014). AR traces were individually analyzed to obtain the mean pharyngeal area (APmean), calculated as the mean of serial measurements of cross‐sectional area of the oropharynx between the incisors and the glottis.

### Sleep and breathing

Sleep was monitored using standard electroencephalograms (C4‐A1, C3‐A2, O1‐A2), electroocculograms, and submental electromyogram (EMG). Leg EMG was also monitored. Breathing was monitored using an oronasal thermistor and pressure cannula during the diagnostic nPSG and using a pneumotachometer during the second night Pcrit study. Abdominal/thoracic excursions were monitored using uncalibrated respiratory inductance plethysmography, and oxygen saturation using pulse oximetry (SomnoScreen Plus, SomnoMedics GmBH, Randersacker, Germany).

Sleep was analyzed according to American Academy of Sleep Medicine recommended scoring criteria (Iber et al. 2007). Hypopneas were defined as ≥30% reduction in airflow, accompanied by ≥4% desaturation for ≥10 sec. An a‐priori apnea‐hypopnea index (AHI) threshold of 10 events/h was used to determine the presence of OSA (Ferrier et al. 2005; Schulz et al. 2007). CHF patients with an AHI ≥10 events/h with >50% of apneas classified as obstructive were classified as having OSA. Patients who had an AHI ≥10 events/h but with >50% of apneas classified as central were excluded from the study.

The AHI in the first half of the night was compared to the AHI in the second half of the night. The study duration was defined as the time between lights being turned off in the evening and lights being turned on in the morning. The study was then divided into two halves of equal duration and the AHI calculated for each.

### Statistical analysis

All data are presented as mean ± SD. Evening and morning measurements of variables were compared using paired‐sample *t*‐tests. Univariate relationships between dependent and independent variables were tested for significance using Pearson product‐moment tests. Statistical significance was determined using a threshold *P*‐value of 0.05. All data were analyzed using R 3.0.2 implemented through R Commander version 2.0‐0 (R Foundation for Statistical Computing, Vienna, Austria).

## Results

Twenty‐eight CHF patients were studied. Of these, five were unable to sleep during the second night of Pcrit measurements and were excluded from analyses. Baseline characteristics of the remaining 23 patients are presented in Table 1. The sleep and respiratory parameters are presented in Table 2. OSA occurred in 13/23 patients (56.5%).

**Table 1:** Participant demographics

Demographics	Mean ± SD
Age (years)	68.3 ± 8
BMI (kg/m^2^)	30.1 ± 5.2
ΔWeight overnight (kg)	−0.8 ± 0.4
Epworth sleepiness scale (/24)	8.5 ± 4.1
NYHA (class I–IV)	2.1 ± 0.6
LVEF (%)	40 ± 14.2
BNP (pmol/L)	54.4 ± 42.8
Cardiac history	
Myocardial infarction (*n*, %)	6 (26%)
Atrial fibrillation (*n*, %)	11 (48%)
Coronary artery disease (*n*, %)	12 (52%)
Dilated cardiomyopathy (*n*, %)	3 (13%)
Medications	
Diuretics (*n*, %)	16 (70%)
B‐blockers (*n*, %)	16 (70%)
ACE‐inhibitors (*n*, %)	14 (61%)

Data are expressed as mean ± SD unless otherwise stated. Although females were not excluded from this study, groups were all males. Patients with diastolic heart failure were not excluded either, however, there were no patients with preserved ejection fraction. BMI, body mass index; NYHA, New York Heart Association; LVEF, left ventricular ejection fraction; BNP, brain natriuretic peptide.

**Table 2:** Sleep and breathing parameters from diagnostic nocturnal polysomnography

Sleep and breathing parameters	Mean ± SD
Respiratory parameters	
AHI (/h)	13.2 ± 11.2
Obstructive apnea index (/h)	3 ± 4.9
Central apnea index (/h)	1.4 ± 2.9
Hypopnoea index (/h)	8.4 ± 6
ODI (4% desaturations/h)	13.7 ± 10.8
Classified as OSA (*n*, %)^a^	13 (56%)
Sleep architecture	
Total sleep time (mins)	372.9 ± 78.9
Sleep efficiency (%)	74.6 ± 12.7
Arousal index (/h)	19.2 ± 14.8
Stage REM (%TST)	19.1 ± 8.4
Stage N1 (%TST)	35.8 ± 19.7
Stage N2 (%TST)	32.3 ± 15.6
Stage N3 (%TST)	12.7 ± 9.3
PLM index (/h)	21.1 ± 25.8
Sleep position	
Supine (%TST)	41 ± 25
Lateral (%TST)	56 ± 28

All data are expressed as mean ± SD unless otherwise stated. AHI, apnea‐hypopnea index; ODI, oxygen desaturation index; REM, rapid eye movement; PLM, periodic limb movement; TST, total sleep time.

a Patients classified as OSA when their AHI ≥10/h with >50% of apneas classified as obstructive.

### Neck circumference and AHI

**Figure 1:**
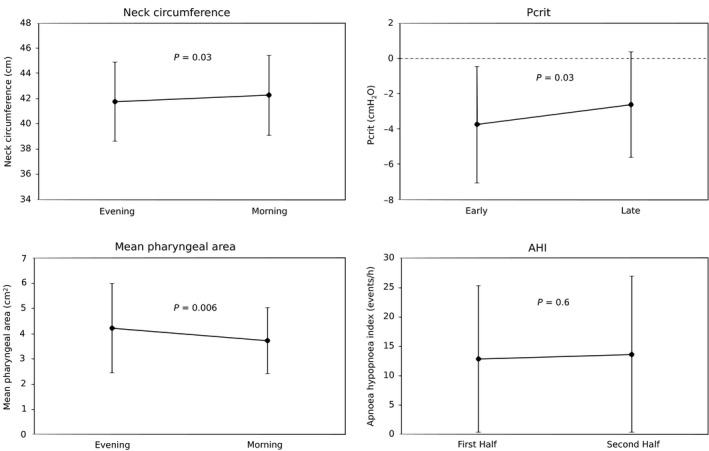
Overnight changes in variables. There were significant overnight changes in all variables except apnea‐hypopnea index (AHI). Evening and Morning measurements of neck circumference and mean pharyngeal area were made during wake, immediately before or after sleep. Measurements of Pcrit were made during non‐REM sleep during the first sleep cycle (Early) and approximately 4 h later (Late). Measurements of AHI were made by dividing the total time in bed into two halves (First Half; Second Half).

Changes in neck circumference are presented in Figure 1. Overall, neck circumference increased significantly overnight (evening: 41.7 ± 3.2 cm; morning: 42.3 ± 3.1 cm; *P* = 0.03). There was an overnight increase in neck circumference in 15 patients, and no increase in eight patients. There was no difference in the AHI between patients with and without an overnight increase in neck circumference (with fluid shift: 14.5 ± 13.2/h; without fluid shift: 10.8 ± 6.1/h; *P* = 0.4). Overall, there was no significant change in AHI between the first and second half of the night (all sleep stages first half: 12.9 ± 12.4/h; all sleep stages second half: 13.7 ± 13.3/h; *P* = 0.6; see Figure 1). This was also the case when the AHI in the first and second half of the night were compared in non‐REM sleep only (non‐REM first half: 11.3 ± 12.9/h; non‐REM second half: 12.4 ± 14.5/h; *P* = 0.3).

### Pharyngeal collapsibility

The overnight change in Pcrit is presented in Figure 1. Pcrit measurements were successfully performed during stage N2/N3 sleep early in the night, and approximately 3 h later during the same sleep stage in 21/23 patients. In the remaining two patients, Pcrit was only measured in the early part of the night. Pcrit increased significantly overnight from −3.8 ± 3.3 cmH_2_O in the early measurement to −2.6 ± 3.0 cmH_2_O in the late measurement (*P* = 0.03), indicating that the pharynx became more collapsible overnight. Early Pcrit measurements were completed 2.1 ± 1.2 h after the start of the sleep study. Late Pcrit measurements began 5.1 ± 1.3 h after the start of the sleep study. The time elapsed between Pcrit measurements early and late in the night was 3.1 ± 1.6 h.

### Pharyngeal caliber

Changes in Pharyngeal caliber (APmean) are presented in Figure 1. Overall, APmean decreased significantly overnight (evening: 4.2 ± 1.3 cm^2^; morning: 3.7 ± 1.3 cm^2^; *P* = 0.006). There was an overnight decrease in APmean in 16/23 patients.

### Correlations between baseline variables and AHI

Exploratory analyses are presented in Table 3. The AHI correlated with Pcrit (*r* = 0.5; *P* = 0.01) and neck circumference (*r* = 0.4; *P* = 0.04), but not with APmean (*r* = −0.3; *P* = 0.1). The Pcrit did not correlate with neck circumference (*r* = 0.04; *P* = 0.8) or APmean (*r* = −0.16; *P* = 0.5) and there was a significant correlation between neck circumference and APmean (*r* = −0.47; *P* = 0.02).

**Table 3:** Univariate correlations of variables at baseline

Dependent variable	Independent variable	Correlation coefficient (*r*)	*P*‐value
Pcrit	AHI	0.5	0.01
	Neck circumference	0.04	0.8
	APmean	−0.16	0.5
APmean	AHI	−0.3	0.1
	Neck circumference	−0.47	0.02
Neck circumference	AHI	0.4	0.04

## Discussion

The aim of this study was to investigate the effects of rostral fluid shift on pharyngeal collapsibility in CHF patients as a mechanism of OSA. The main finding was that neck circumference increased overnight, indicating fluid shift, and this was accompanied by an overnight increase in pharyngeal collapsibility measured during sleep, and decrease in pharyngeal caliber. There was a significant correlation between AHI and Pcrit, and AHI and neck circumference, however, there was no significant overnight change in the AHI.

### Overnight changes in pharyngeal collapsibility

To the best of our knowledge, this study is the first to measure Pcrit in CHF patients during sleep, and the only study to have made overnight measurements during early and late non‐REM sleep. Our primary hypothesis was confirmed by the finding that Pcrit significantly increased overnight. This finding supports the notion that passive overnight fluid shift acts directly on the pharynx to increase its propensity to collapse. Previous studies have relied on actively inducing acute fluid shift to increase Pcrit during wake (Su et al. 2008). Here, we have translated these data into a clinical context by studying CHF patients with and without OSA during sleep.

Pharyngeal collapsibility is known to correlate with the AHI in the general population (Kirkness et al. 2008) and it is higher in OSA patients than healthy controls (Patil et al. 2007). A Pcrit of −5 cmH_2_O has been found to be a critical threshold in the general population; sleep apnea is rare in people with a Pcrit less than −5 cmH_2_O but is increased markedly in people with a Pcrit greater than −5 cmH_2_O (Kirkness et al. 2008). In this study, the mean Pcrit was greater than the critical −5 cmH_2_O and correlated significantly with the AHI. Therefore, we conclude that Pcrit measured during non‐REM sleep is associated with the severity of sleep disordered breathing in CHF patients in a similar way to the general population.

### Overnight changes in pharyngeal caliber

A significant overnight decrease in APmean confirmed our secondary hypothesis. Previous studies have demonstrated an association between pharyngeal caliber and actively induced acute rostral fluid shift in healthy volunteers (Chiu et al. 2006; White et al. 2014). The results of this study extend these findings to demonstrate an association between passive overnight rostral fluid shift and pharyngeal caliber in CHF patients with and without OSA.

In the general population, there is an association between a narrow pharyngeal caliber and OSA (Ciscar et al. 2001; Dempsey et al. 2002). Our group has also previously shown that older age may exacerbate anatomical risk factors for OSA, and conversely, that a larger pharyngeal caliber is protective against OSA in older age (Carlisle et al. 2014). This is relevant for CHF patients because the prevalence of CHF increases markedly with advancing age (Scarborough et al. 2011). The current data suggest that rostral fluid shift to the neck acts directly to reduce pharyngeal caliber across the night in CHF patients. This may exacerbate age‐related anatomical changes to the pharynx that predispose people to OSA in older age.

### Overnight changes in neck circumference

The overnight increase in neck circumference was consistent with previous studies of OSA patients and CHF patients (Redolfi et al. 2009; Yumino et al. 2010; Fischer et al. 2012). As in previous studies of OSA patients (Jafari and Mohsenin 2011), not all of the CHF patients in this study experienced fluid shift. Other researchers have speculated that not all lower leg fluid shifts to the tissues of the neck, and that some may be redistributed to other body compartments such as the abdomen and the thoracic cavity (Yumino et al. 2010; Kasai et al. 2012). Unfortunately, we were not equipped to make measurements of fluid volume in the lower legs and other body compartments in this study.

### Overnight changes in AHI

We found no significant change in the AHI between the first and second half of the night, which is consistent with one other study of OSA patients without CHF (Jafari and Mohsenin 2011). Others have suggested that the lack of an overnight change in AHI may be because the majority of fluid shift occurred quickly upon assuming a recumbent posture (Kasai et al. 2012). The notion that fluid shift is rapid and posture dependent is supported by evidence that neck circumference is increased and ankle circumference is decreased when moving from the upright to supine posture (Fischer et al. 2012). However, the same study also showed a further increase in neck circumference and decrease in ankle circumference overnight, suggesting that there is a delayed or slower overnight fluid shift. We also found evidence of a delayed or slow overnight fluid shift since our Pcrit measurements started approximately 2 h into the sleep study and showed an increase in Pcrit approximately 3 h later. If the change in Pcrit was attributable to fluid shift, this would suggest that not all fluid shift occurs rapidly at the start of the night. Future research to investigate the time course of overnight fluid shift may be of value in phenotyping patients and targeting treatments to minimize the impacts of fluid shift on OSA severity.

The findings of our study suggest that the AHI is an insensitive measure of fluid shift. The AHI during the first and second half of the night could be affected by a number of confounding factors. We attempted to control for differences in the distribution of sleep stages by also comparing non‐REM AHI. However, other factors such as changes in arousal threshold, respiratory drive, and body position were not controlled. Furthermore, exploratory analysis showed no difference in OSA severity when patients were subdivided into those with and without overnight fluid shift (indicated by an overnight increase in neck circumference).

A number of associations between variables measured at baseline are consistent with previous research, and provide external validity to this study. There was a significant correlation between Pcrit and AHI which is consistent with a larger study of the general population (Kirkness et al. 2008); neck circumference correlated with the AHI which is consistent with previous studies of OSA patients and CHF patients (Redolfi et al. 2009; Yumino et al. 2010); the APmean correlated with neck circumference, which has been noted before in healthy volunteers (Shiota et al. 2007).

## Limitations

A technical limitation of the study design was that we sought to measure the effects of rostral fluid shift during sleep, early and later in the night, using the Pcrit technique, which may in itself have disturbed sleep, and potentially may have attenuated rostral fluid shift. Additionally, therapeutic levels of CPAP have been shown to attenuate fluid shift in CHF patients with OSA (Yumino et al. 2010) and CPAP near to or at therapeutic levels are required for the holding pressure in the passive Pcrit technique. In order to allow as much overnight rostral fluid shift to occur as possible, we only supplied CPAP at holding pressures during the actual Pcrit measurements. In the intervening hours between early and late Pcrit measurements, we turned CPAP down to the minimum pressure sufficient to prevent CO_2_ accumulation in the nasal mask (~3 cmH_2_O).

## Conclusion

This study supports rostral fluid shift as a factor leading to increased collapsibility of the pharynx in CHF. We have shown that there is likely to be a “slow” overnight component to rostral fluid shift in addition to the “fast” component that has been observed previously, either by inducing acute fluid shift with lower body positive pressure, or immediately after assuming a recumbent posture. Our findings suggest that simple overnight measurements of neck circumference may be a useful clinical measure for CHF patients who are susceptible to rostral fluid shift.

## Conflict of Interests

None declared.
